# A211 CAN AN EXPERIMENTAL MOUSE MODEL BE USED TO CHARACTERIZE BEHAVIOURAL COMORBIDITIES ASSOCIATED WITH INFLAMMATORY BOWEL DISEASES?

**DOI:** 10.1093/jcag/gwac036.211

**Published:** 2023-03-07

**Authors:** J Josephson, J Barnett, H Chiang, D Gibson

## Abstract

**Background:**

Inflammatory Bowel Diseases (IBD) are increasing globally with westernized countries experiencing a dramatic increase in cases of ulcerative colitis and Crohn’s disease. The corresponding rise in the comorbidities of anxiety and depression are linked to inflammation and microbiome dysbiosis; impacting the gut-brain axis (GBA). Another facet of the GBA are the tryptophan metabolites serotonin (5HT) and kynurenine (Kyn). 5HT and Kyn are metabolites implicated in anxious behaviours and memory deficiencies with dysbiosis influencing metabolite production. In inflammatory conditions, upregulation of Kyn impacts brain homeostasis as Kyn has both positive and negative brain metabolites. As disease outcomes are tied to the severity of depression/anxiety, characterization of the behavioural and tryptophan metabolite profile of an IBD model can allow us to develop better IBD therapies.

**Purpose:**

To characterize the anxiety and memory phenotype with corresponding assessment of tryptophan metabolites 5HT and Kyn across tissues of interest in an spontaneous colitis model.

**Method:**

Mucin 2 knockout mice (Muc2^-/-^) lack the protective mucin 2 layer in the distal colon, allowing for direct contact between the gut contents and the gut epithelial layer. Over time this contact results in the development of spontaneous colitis. Muc2^-/-^ n=12 per sex were compared to C57Bl/6 mice for behaviour and tryptophan metabolites across brain, colon and serum. Open Field Test and Light/Dark tests were used to assess anxiety levels, Novel Object and Radial Arm maze were performed to assess memory deficits. Mice underwent one anxiety and one memory test and were euthanized following their last maze. Enzyme-linked immunosorbent assay (ELISA) assessment of 5HT and Kyn were performed on brain, colon and serum samples. T-tests were performed and graphs created using PRISM (v9.4.1).

**Result(s):**

Muc2^-/-^ mice had decreased anxiety (p=0.0113) compared to C57Bl/6 wild type mice in the light/dark maze. Muc2^-/-^ displayed no significant differences in novel object preferences or the number of memory errors in novel object recognition test and radial arm maze respectively. Serum kynurenine was found to be significantly elevated (p=0.0444) in Muc2^-/-^ mice. The sex factor has to be mentioned here in the results not in the conclusions.

**Conclusion(s):**

IBD is increasing globally and problems with mental health is often comorbid. Within our model we were able to show that behavioural and biochemical differences do exist between our Muc2^-/-^and wild-type mice with depressed anxiety responses and heightened serum kynurenine, however the implications of these findings require further investigation. Understanding how inflammation is driving behavioural alterations can provide pathways to ameliorate the behavioural comorbidities in IBD patients suffering from anxiety of memory deficits.

**Disclosure of Interest:**

None Declared

